# Narrative Deference

**DOI:** 10.1007/s11245-024-10105-z

**Published:** 2024-09-26

**Authors:** Eleanor A. Byrne

**Affiliations:** Department of Philosophy, University of Birmingham, Birmingham, UK

**Keywords:** Self-narrative, Distributed cognition, Memory, Trauma

## Abstract

Recent work on distributed cognition and self-narrative has emphasised how autobiographical memories and their narration are, rather than being stored and created by an individual, distributed across embodied organisms and their environment. This paper postulates a stronger form of distributed narration than has been accommodated in the literature so far, which I call *narrative deference*. This describes the phenomena whereby a person is significantly dependent upon another person for the narration of some significant aspect of their own autobiographical self-narrative. I suggest that a person is more likely to narratively defer where they suffer a mnemonic impairment regarding some significant adverse life experience like trauma, illness or injury. Following a recent turn in the literature towards investigating the harmful aspects of distributed cognition as well as its many advantageous features, this paper explores how the benefits of autobiographical self-narrative deference within close personal relationships are complexly related to its harms.

## Introduction

Research in situated and distributed cognition and affectivity has long-emphasised the role of things and people in our environments for the cultivation of various cognitive and affective capacities and states in us. Much of this research focuses on the positive aspects of what such resources can afford us: for instance, cognitive scaffolds allow us to realise certain cognitive functions, which in turn helps us realise certain goals (Clark 2006); other people help us to regulate our emotions (Varga and Krueger 2013); evocative objects can scaffold affective states like joy, help us to regulate our emotions and support a healthy emotional life (Colombetti and Krueger 2015). Conversely, some research has illustrated how the *privation* of various resources can have negative or harmful effects on our cognitive and emotional life, such as lack of social goods in chronic illness and loneliness (Byrne 2024; Krueger et al. 2023).

It is not only privation that can cause harm. Of course, things and people can have active influences on us that are harmful. Recently, there has been a wave of interest in explicating on what grounds we can understand varieties of active engagement with scaffolds to be harmful, for instance: how we distinguish pernicious *mind invasion* from the broader category of mind *shaping* which can also be decidedly positive, such as in cognitive bias modification therapy and positive instances of “nudging” (Walter and Stephan 2023); the ways in which organisms can modify features of their environment in ways that are harmful to them (Coninx 2023); and how public statues can function as deeply harmful affective artefacts (Archer 2024).

In this paper, I focus on how we use other people to help us construct our own autobiographical self-narratives. The distributed nature of our self-narratives has been discussed extensively in recent years (Menary 2008; Heersmink 2017, 2018, 2020; Fabry 2018, 2023a, b, c). However, what has not yet been adequately captured is just how strong this dependence can be – that is the gap I intend to fill here. Often, the memories that others help us retrieve are fairly trivial and narrow in scope: I might ask my colleague to remind me what time a meeting begins to be sure that I don’t arrive at the wrong time, or ask an old classmate what year we were awarded our degree so that I can fill out a job application correctly. Sometimes, the role of other people in supporting memory retrieval is much more significant and broader in scope. For instance, somebody may enlist a parent or a sibling to help them remember certain childhood events, or a romantic partner to help them remember things about their experience of significant injury or illness. In these sorts of cases, such events might be autobiographically significant to the person such that the memories others can fill in significantly shapes how they narrate their life and identity. We can understand such features of one’s autobiographical self-narrative as distributed since they have been written in collaboration with others.

In this paper, I draw attention to a particularly strong form of autobiographical co-narration which I will call *narrative deference.* If I narratively defer to somebody, I have afforded greater narrative authority to them relative to myself over an experience of my own which is significant for my autobiographical self-narrative. In what follows, I will specify how narrative deference operates, and suggest conditions for it, before drawing attention to its potential harms. The structure of the paper will be as follows. In Sect. 2, I run through extant work on distributed nature of memory and autobiographical self-narrative. In Sect. 3, I draw upon this body of literature to develop the notion of narrative deference. In Sect. 4, I expand on some background conditions under which narrative deference can appear as possible and desirable. In Sect. 5, I show how dynamics of narrative deference can give rise to a variety of harms and injustices.

## Memory and Self-Narrative

The position that cognition is distributed takes it that human cognitive systems, like memory systems, are distributed across embodied agents and environmental structures such as artifacts and people (Hutchins 1995; Michaelian and Sutton 2013). Tracing back to Wegner’s work in the 1980s on transactive memory systems, researchers have studied how memories can be intersubjectively supported. The central tenet of a transactive memory system is that “group memory structures develop and become capable of memory feats far beyond those that might be accomplished by any individual” (Wegner et al. 1985, p.319). More contemporary research on the distributed nature of memory systems has identified interesting connections between memory and cognitive interdependence. For instance, some empirical research on memory recall in long-term married couples found that in general, memory recall is higher when couples are seated together than when they are alone (Sutton et al. 2010; Harris et al. 2014; Sutton 2018). In one such study, a participant commented that her and her husband could “each tell the other’s stories”. Later, her husband expanded “…because we’ve done everything together…if you’re not careful you don’t know who has the right to the story” (Harris et al. 2011, p.294). The picture that these data give us is that those we are in close relationships with shape and influence the processes of accessing, interpreting and recalling our autobiographical memories.

Heersmink has extensively explored the connection between the distributed nature of memories and of narratives, and has argued that our narrative identities are distributed given that the memories that scaffold them are distributed (2020). He argues that we remember our past by interacting with things and people in our environment, that is, the stuff makes up our *memory ecology*. Our memory ecologies are made up of evocative objects and transactive memory partners, i.e. the group of people that make up a transactive memory system. He takes the notion of evocative objects from Turkle, who characterises evocative objects as extra-neural objects that generate some cognitive or affective response in us (2007).^1^

Heersmink understands our attempts to manage our memory ecologies as a form of cognitive niche construction (2020). Where niche construction theory more generally looks at how organisms actively alter their environments, cognitive niche construction refers to how animals manipulate their environments and create cognition-aiding tools in order to support their functioning (Sterelny 2005; Clark 2006). Here, we can understand the act of engaging with evocative objects as supporting certain cognitive processes, namely the construction and retrieval of autobiographical memories. Heersmink is interested in emphasising the distributed, cultural and interpersonal dimensions of these ecology-engineering processes, and in so doing identifies three types of niche construction processes that govern how we interact with our memory ecology: creating, editing, and using (2020, p.15).^2^ In *creating* a resource for my memory ecology, I might take a photograph of an event or buy a piece of art to remind me of a holiday with the intention of making these artefacts available to me later to retrieve and construct a memory of those events.

Of course, an object can remind me of an event without any such intention on my part. A piece of music might remind me of a particular period of life, for example. Such features of my memory ecology have not been intentionally engineered by me, yet once I am aware of the association I can later engage with the artefact more intentionally by, say, scaffolding certain emotions by listening to certain musical pieces.^3^ This is now in the sphere of *editing* i.e. intentionally altering how my memory ecology is set up. We might also change how accessible a material evocative object is to us by throwing it away or storing it differently (like putting letters in a box in the attic). Thirdly, we may *use* our memory ecology by interacting with the artefacts in our memory ecology as engineered by us. This might include initiating a conversation with a family member to discuss their memory of certain key events that matter to us.

Heersmink subscribes to the view that our identities are *constituted* by our autobiographical self-narratives; that is, the narrative self-constitution view (Schechtman 1996, 2005; Bruner 2003).^4^ According to Heersmink, selfhood is constituted by subjective, affective and personal stories made up of (mostly) accurate chronological description of a series of connected events and experiences. Heersmink sought to integrate the narrative self-constitution view with the distributed memory thesis by moving from the claim that the memories that constitute the “building blocks” of our narrative identity are distributed, to the claim that our narrative identity as a whole is distributed (2020).^5^

Fabry rejects Heersmink’s account of why self-narratives are distributed because she takes Schechtman’s view, on which Heersmink relies, to be incompatible with the wider distributed cognition framework (2023b). Fabry locates the problem in later, qualified versions of Schechtman’s view where she postulates the ‘articulation constraint’. This constraint qualifies her earlier claim that self-narratives are the products of “implicit organising principles” within the mind. Later, Schechtman added that the products of these implicit organising principles must be at least *articulable*, though not necessarily articulated (Schechtman 2005). Fabry criticises this view on the grounds that it takes an internalistic and individualistic view of self-narrative, where the narrative is produced and stored internally, before and regardless of its articulation. This, she contends, is incompatible with the distributed cognition framework which emphasises how other human organisms and cognitive tools play an important functional role in the realisation of many cognitive processes.

Fabry does not abandon the claim that self-narratives are distributed. Instead, she wants to follow the distributed cognition framework to emphasise how socio-cultural conditions enable the construction of self-narratives. She writes “we actively establish connections between distributed autobiographical memories in virtue of our engagement in distributed self-narrative practices” (2023b, p.1259). In what follows, I follow Fabry (2023b) in understanding self-narrative as the self-referential narrative representation of autobiographically remembered past events or experiences, where these memories are shaped by the narrating person’s interaction with other people and/or things, such as evocative objects or members of a transactive memory system (ibid.).^6^

Something that is important for Fabry’s account is that the configuration of autobiographical memories is influenced by the narrative activities of the “narrating I”. She writes: “It is the narrating I that selects, connects, contextualises, and interprets autobiographically remembered personal past events or experiences.” (2023b, p.1266).^7^ She takes this to be a critique of Heersmink’s view of (distributed) memories as the “building blocks” of (distributed) self-narrative: on her view, memories are not like bricks which are later used to build narratives; rather, memories are plastic components that dynamically influence the narrative process (ibid.). This clarification invites clearer consideration of how the configuration of autobiographical memories and self-narratives are significantly shaped by narrative templates and social scripts (Ratcliffe and Byrne 2022), as well as subtler linguistic and paralinguistic gestures by interlocutors (Norrick 2007; Fabry 2023b).

In her discussion of spontaneous conversational self-narrative, Fabry follows Norrick (2019) and acknowledges that where experiences are shared between the narrator and the interlocutor, the interlocutor might contribute semantic or episodic details that influence the self-narrative process (2023b). As such, both Norrick and Fabry have proposed that we understand the distributedness of conversational self-narrative as existing on a continuum (Fabry 2023b, p.1267). Sutton (2018) has noted that in distributed memory systems, cognitive interdependence does not have to be “merger”, but instead is often complementary. This means that each member of the group does not contribute the same memories, but that each member offers a more or less subtly different contribution such that the overall contribution of the group is broader. Given the tight connection between recalling and narrating memories, one would expect narrative contributions to mirror this asymmetry. While acknowledging that more fine-grained distinctions can be made, following Norrick and Fabry we can distinguish between three broad levels of distributed self-narrative: (1) weak distributedness: here, interlocutors contribute to the self-narration process by offering opportunities for refinement, elaboration, or the re-interpretation of autobiographically remembered events or experiences (2) moderate distributedness: here, interlocutors might contribute substantial semantic or episodic details to the narrative and (3) strong distributedness: here, interlocutors collaboratively narrativise autobiographical memories to form a “single narrative thread” (Norrick 2019).

For Norrick, co-narration of this strength requires that speakers share “telling rights”, which he takes to be an epistemic criterion for an entitlement to tell a story and impart knowledge of that story onto others. One has telling rights either through direct epistemic access to the events narrated or some other sort of “insider” knowledge like being told about an event in detail (2019). Fabry notes that at the ‘extreme’ end of this continuum, we might no longer be able to clearly distinguish between transactive memory systems and transactive narrative systems since the retrieval of autobiographical memories and self-narration are so tightly interwoven (2023b p.1267). In what follows, I want to go look more carefully at the strong end of this continuum to show that distributedness of this degree is much more widespread than the extant literature would suggest. These sorts of cases put pressure on Fabry’s idea that “It is the narrating I that selects, connects, contextualises, and interprets autobiographically remembered personal past events or experiences.” (2023b, p.1266).

## Mnemonic Impairment and Narrative Deference

In what follows, I explore cases of particularly strong distributed narration through the lens of a particular subset of events, namely significant adverse life events that involve significant memory impairment. There are various reasons why we might not remember adverse life events particularly well, after accommodating for the fact that “normal” well-functioning memory is already prone to distortion (Schachter et al. 2011; Michaelean 2021). Memory can also be significantly affected by factors like age, illness, injury and trauma. While views about the “childhood amnesia” period differ, it is uncontroversial that very young children cannot access episodic memories of early life events as consistently as adults (Hayne and Jack 2011; Bauer 2015; Peterson 2021). Likewise, there is robust and varied evidence showing how particular illnesses and injuries involve (either as a symptom of the condition or its treatment) significant memory and/or attention impairment, such as epilepsy (Butler and Zeman 2008; Novak et al. 2022), psychosis (Seabury and Cannon 2020), brain injury (Jolly et al. 2020) and other medical emergencies requiring intensive care (Jones et al. 2000; Murray et al. 2020).

A vast body of research illustrates how people with trauma and stress-related disorders have significantly impaired memory of the event(s) resulting in corresponding narrative disorganisation compared to controls (Herlihy et al. 2002; Jones et al. 2007; Jelinek et al. 2009; Salmond et al. 2011; Brewin 2016; McGuire et al. 2021). Famously, this impairment has been associated with dissociation during exposure which affects how memories are encoded, resulting in retrieved traumatic memories being experienced as “mental imprints of sensory and affective elements of the traumatic experience” involving visual, olfactory, affective, auditory, or kinesthetic elements (van der Kolk and Fisler 1995, p.505). How the memories are encoded and processed can also result in difficulty contextualising the memory against events that happened before or after the traumatic event:

If the individual lacks conceptual processing and engages mainly in data-driven processing (i.e. processing the sensory impressions), then the trauma memory will be relatively difficult to retrieve intentionally and at the same time there will be relatively strong perceptual priming for accompanying stimuli […] The resulting memory trace will be poorly discriminated from other memory traces (Baddeley 1997), thus impairing stimulus discrimination between stimuli present during the trauma and harmless stimuli that bear some similarity to these. (Ehlers and Clark 2000, p.331)

Moreover, these influences are commonly comorbid with one another in ways that can exacerbate memory impairment. For instance, trauma occurs in childhood (Chu et al. 1999; Hughes et al. 2017),^8^ trauma can be caused by illness, injury or its treatment such as PTSD after intensive care (Parker et al. 2015), trauma can cause later pathology later in life (Danese 2020) and various conditions which involve memory impairment are commonly comorbid or complexly related such as psychogenic seizure, trauma, psychosis and epilepsy (Chohan et al. 2023).

Other people can be involved in these experiences to varying degrees.^9^ At the strong end of the scale, in instances of trauma, a child and their parent may have been abused together (like in cases of “wife and child” abuse; see Tajima 2004). In cases like these, the child’s mother may have both directly witnessed the traumatic event(s) and its developmental effects on the child over time. Still, in cases where the parent did not directly witness the traumatic events, they may still have known propositionally, to varying degrees, what has happened and witnessed its effects on the child over time. Similarly, a partner or family member may have been closely involved with the full trajectory of a person’s illness or injury—from prodromal period to diagnosis to treatment to recovery—as well as the broader context of their life such as aetiological factors.

Other significant adverse experiences may have been experienced alone, but other people, such as a romantic partner or a therapist, become significantly epistemically informed about the experiences some time later. For instance, a person who has been assaulted earlier in life may inform their partner or a therapist about this event many years later, such that their partner or therapist becomes highly indirectly acquainted with the events and is able to contextualise the event alongside the person’s current behaviour.

On Norrick’s (2019) account of telling rights, “B-people”—parents, friends, partners, therapists—can qualify as having telling rights over A-experiences because of their direct or indirect acquaintance with the events in question. With this in mind, I now want to consider a stronger form of co-narration where, stronger than instances where interlocutors collaboratively narrativise autobiographical memories to form a single narrative thread, narrative authority over A’s experiences which matter for their autobiographical self-narrative is afforded to B. This is what I call *narrative deference.* I suggest that narrative deference is significantly more likely in cases where there is a mnemonic impairment over some significant adverse life experience, though it is neither necessary nor sufficient for it. I will say more about this and other background conditions for narrative deference in Sect. 4, but for the remainder of this section, I will continue to sketch out how narrative deference might look.

Narrative deference involves strong narrative *asymmetry*. If A narratively defers to B, is not the case that person A and B are collaboratively narrativising some experience of A’s, but rather, B takes narrative authority relative to A over some experience of A’s which is central for her autobiographical self-narrative. Narrative asymmetry is not sufficient for narrative deference, however. There may be some asymmetry in details remembered, and minimal deference to specific facts which reflect those asymmetries. This is recognised as a typical feature of distributed memory systems (Sutton 2018). This means that each member of the party does not contribute the same memories, but that each member offers a more or less subtly different contribution, such that the overall contribution of the group is broader; this is a key reason that distributed memory systems are so useful.

On the other hand, consider the following cases:

1. A boy, whose father assaulted him in front of his mother when he was a young child. He has few memories of this and employs situational management to avoid “unlocking” them. As a young adult, he asks his mother more questions about what happened, and how she believes the abuse has subsequently affected his behaviour and character. He does not remember the events that his mother describes, but they have a quality of “making sense” and he trusts their veracity and adopts the information to help him navigate his current relationships. Some combination of trauma and age may underlie this mnemonic impairment.
2. An adult woman suffers from functional seizures. She is dependent upon her partner for details about her behaviour to know when a seizure is likely to come; she often dissociates beforehand and lacks insight into when this is happening. She also depends upon her partner’s judgement to interpret and make sense of her feelings before, during and after her seizures, which influences how she reports to her psychiatrist. Illness, and perhaps comorbid psychopathology or trauma, underlies this mnemonic impairment.

Cases like these involve willing narration of A’s significant experience(s) *through* the epistemic standpoint of B which is taken to be epistemically stronger than A’s. This is significantly qualitatively different from collaborative memory recall with roughly symmetrical epistemic telling rights. For these individuals, the recall and narration process is not equally distributed across the dyad. Rather, B is attributed narrative authority over some aspects of A’s own experience. In this sense, A’s autobiographical self-narrative is significantly—perhaps even predominantly—influenced by co-narrator B. Where there is narrative deference, then, A is more dependent upon B for central aspects of A’s autobiographical self-narrative than on (A) Of course, this is hard to judge, can come in degrees, and is not static through the course of a relationship. In cases where this dependence is particularly strong, it may be that A feels as though a narration of some event X just would not be possible at all without (B) Or perhaps a narration of some event X, if attempted by A alone, would have a felt quality of having been “made up”, and hence fail to satisfy epistemic criteria required for endorsement: A *could* be the sole narrator of the experience, but may be unable to trust or commit to it.

## Instantiation and Expectation

We have already distinguished between degrees of distributedness of autobiographical self-narratives, generally with a focus on spontaneous conversational narrative. With the notion of narrative deference identified as the strongest class of distributed autobiographical self-narration, within this we can also distinguish between different strengths of narrative deference. At one end of the scale would be fairly trivial instances of narrative deference: whilst one’s autobiographical self-narrative is always distributed to some extent, minimal narrative deference is also a typical feature of our close interpersonal relationships throughout our lives. For instance, one might narratively defer to a trusted individual on a particular occasion where one’s mnemonic resources regarding that event are anomalously limited. For instance, somebody might narratively defer to a family member about things they said while being sedated before an operation, or to a friend about a psychedelic experience. Such events might be positively or negatively valanced, and more or less integral to one’s unfolding autobiographical self-narrative.

Some more moderate forms of narrative deference I also take to be fairly typical. In his discussion of “downstream narrative niche construction”, Heersmink (2020) suggests that since our caregivers are a natural place to turn to learn about earlier periods of our lives, we can understand intergenerational transactive memory systems as significant scaffolds for our narrative identity. Empirical study appears to support the position that parent-child, and especially mother–child, dyads are often particularly salient intergenerational transactive memory systems in which the mother functions as the autobiographical memory system in childhood and adolescence, thereby scaffolding the content of the child’s narrative as well as the narrative skills that they can employ to narrate experiences later in life. Indeed, Fivush et al. (2011) offer evidence that children whose parents structured more elaborate and coherent autobiographical narratives with and for them, show a more coherent sense of self later in life (Fivush et al. 2011; Heersmink 2020). Since memories of early childhood are fragmented and sparse, we might consider the caregiver the dominant narrator in such exchanges such that the child is in a deferential position. Similarly, these events or periods may be positively or negatively valanced, and more or less integral to one’s unfolding autobiographical self-narrative.

Something that is overlooked in these discussions is that the process of creating and narrating childhood events is often a life-long one: it does not stop after childhood or adolescence. While a significant part of our autobiographical self-narrative is scaffolded by our caregivers in early life, it is also important to note the life-long nature of this process. As illustrated by case (1) above, the desire to enlist our caregivers to help us to create, reinterpret or recontextualise memories and narratives of early life experiences can arise in response to a variety of later life experiences. Such experiences might include grief, trauma, illness, relationship conflict, worsening phobias, and other consolidated behaviours that become maladaptive later in life.

Of course, it is not the case that everybody can or wants to enlist a caregiver or parent to co-author aspects of their autobiographical self-narrative. There are many reasons for this, including but not limited to, the closeness and accessibility of the relationship. Perhaps one has been estranged from one or both parents, or perhaps they have died or significantly changed due to cognitive impairment in illness. In cases like these, it may still be that the caregiver *would* or *should* have possessed the relevant narrative material, but they are no longer accessible. It may be that one wishes to enlist a parent to narrate and contextualise their grief response to that very parent’s death (Ratcliffe and Byrne 2021). With these considerations in mind, it becomes clear that a full appreciation of narrative deference requires an analysis not only of its instantiation in terms of active narrativizing, but also the background structures that make narrative deference feel possible, desirable or necessary.

### Defying Narration

I do not wish to claim that one *only* narratively defers about experiences which defy one’s own narration, however, it is certainly a predisposing factor. To clarify what it means for an experience to defy narration, I draw on Køster’s scalar approach to narrative embodiment (2017) which attempts to articulate the complex relation between self-narrative and other deeper more embodied dimensions of self. On this account, he distinguishes between three categories of experience: unnarratable; narratable; and narrative. The unnarratable refers to a level of embodied experience that evades narrative order, or at least cannot be *adequately* captured by narrative. Following Waldenfels (2002), Køster takes the *passive* dimension of embodied experiencing to explain this, for this involves layers of embodied meaning that evade full narrative configuration.^10^ He offers trauma, seizure, and certain aspects of falling in love as examples of experiences whose full embodied meaning evades narrative order.

Narratable dimensions of experience, on the other hand, are amenable to narrative order but have not yet been made into narratives: they are, On Køster’s account, “eligible for being brought to narrative integration through various degrees of effort” (2017, p.901). Køster appeals to Waldenfels’ (2000) notion of the *split self* to point to this dual character of some embodied experiences whereby the internal and external perspective of the body are both salient. This, he writes, “can be the basis for a range of experiences of alienness and unfamiliarity: of what belongs to the familiar order of my self-acquaintance and what evades that order” (2017, p.897). There can be more or less mundane experiences with this character. Køster mentions the phenomenon of waking up to find one’s arm “dead” having been sleeping in such a way that restricts blood flow. We can also take some psychopathological experiences, such as dissociative experience, to take this form. Writer Jules Evans, who has written and spoken extensively about how Ancient Greek philosophy helped him to recover from early adulthood mental illness, describes in a TEDx talk not knowing when his social anxiety would “jump out and humiliate” him (Evans 2013). The embodied feelings of anxiety are surely his—and felt deeply as such since they persecute and humiliate *him*, nobody else—but also have a character of being a foreign invader, exerting control over him at their own whim.

Consider other recurring embodied experiences such as the seemingly meaningless “jumpiness” of a hypervigilant nervous system, or waves of deep anger when light-heartedly teased. Such patterns may be the result of trauma in response to experiences which are not well-remembered. Such experiences are embodied signatures of our personal past which can be understood as “sedimented” body memories, even if they are not remembered episodically (Colombetti and Bogotá 2024). Recall the empirical research showing that trauma memories are more likely to have been processed as sensory impressions, and thus often have a more sensory character: this diffuse sensory character makes it harder to distinguish between harmful and safe stimuli, leading to more diffuse triggers (van der Kolk and Fisler 1995; Ehlers and Clark 2000).

Without being given meaning through narrative, experiences like these may have a threateningly alien character. Køster discusses psychotherapy as an example of a process which can lend narrative form to certain confusing experiences—narratable but not yet narrated—which still “belong to” and exert control over oneself. Giving narrative form to such embodied experiences can cultivate a felt sense of agency over dispositions and character traits “that would otherwise control me from “beyond”” (Køster 2017, p.906). I suggest that as well as therapists, other people we are close to in our lives can also play this role for us by contributing certain epistemic, mnemonic and narrative resources.

For instance, in Case 1 discussed above, the victim of childhood abuse may find himself to be made disproportionately irritable and anxious when exposed to certain sounds in ways that he cannot make full sense of. He may find it plausible or even suspect that it has roots in his abuse, but not feel equipped to construct a narrative worth giving credence to. In lieu of the required resources, he may turn to his mother to contextualise these bodily feelings and give them narrative order. It may be the case that his mother is experienced as being the *only* person who can bring narrative order to experiences and feelings like these. In knowing that she is in possession of mnemonic and epistemic resources about certain events that he lacks, he may experience her as literally possessing parts of him.

### Trust

So what background structures are necessary for narrative deference? It may *prima facie* seem that being able to turn to a particular person to give narrative order to confusing, alienating experiences is inherently positive. Strengthening one’s autobiographical self-narrative, self-understanding and sense of agency are all valuable, as well as being valuable features of enjoying close interpersonal relationships. Hence, one might assume that the very possibility of narrative deference to another presupposes a positively valanced dyadic relation. For instance, one might assume that a necessary condition for narrative deference is that one trusts the person whom they narratively defer to. I argue that while trust is necessary for narrative deference, it only need be a very thin notion of trust.

Currently, it is left ambiguous what structural features of a relationship are required for narrative deference, and why having a desire to give narrative order to certain confusing experiences is generally not sufficient for us to take on all of the narrative contributions that our friends, family members, colleagues and therapists offer about us. Suppose that A is reflecting on an event in their childhood with their relative B. B may offer her judgement of how that event influenced A’s character which conflicts A’s. There might be good epistemic reasons to endorse B’s narrative, if, for example, B was an adult at the time and are more likely to remember the experience than A, yet it may still not stick.

We must appeal to the notion of trust to explain the complexity at play here. A number of philosophers have emphasised the fundamentality of trust in our interpersonal interactions. Løgstrup, for instance, takes trust to be built into how we anticipate and encounter others (1956/1997). This trust is characterised by an openness to the other and to having one’s world shaped by them: “By our very attitude to one another we help to shape one another’s world” (1956/1997, p.18). Ratcliffe comments that if one endorses something like this view, then “trusting anticipation is not just one of the ways in which we encounter others—it is inseparable from the potential to be affected by someone in a specifically personal way.” (Ratcliffe 2024, p.12). What particular people we trust, and exactly what we trust them for, is contingent. However, on this view, there is a more basic, fundamental form of trust which is not: “Trust is not of our own making; it is given. Our life is so constituted that it cannot be lived except as one person lays him or herself open to another person and puts her or himself into that person’s hands either by showing or claiming trust” (Løgstrup 1956/1997, p.18). I take this to draw attention to a minimal, background sense of trust that makes us vulnerable to more or less subtle influences from others, regardless of how and whether we trust them in more propositional ways (e.g. “I trust A to do B for me”). Similarly, Govier has argued that trust is a basic condition of our having any experience at all: “We need trust to observe and remember events in the world and to maintain knowledge of ourselves. Such trust can be argued to be a priori because there is a sense in which it is logically prior to experience itself.” (1993). Later she writes “without trust, personal and social life would be impossible.” (1998).

As well as trusting other people, another important dimension of trust is self-trust. Govier takes self-trust to be a necessary condition for having self-respect and autonomy (1993). She suggests that memory impairment can significantly undermine self-trust, and explicitly discusses mnemonic impairment in childhood as particularly destructive: “To lack general confidence in one’s own ability to observe and interpret events, to remember and recount, to deliberate and act generally, is a handicap so serious as to threaten one’s status as an individual moral agent” (p. 108). With this in mind, I suggest that a fuller understanding of the mechanisms behind narrative deference for any relationship, requires specification of (i) the domain of trust and (ii) its strength in that domain relative to trust in oneself. In so doing, we can make sense of how narrative deference can operate in relationships that are acrimonious, infected with suspicion, resentment, indignation and—apparent contradiction notwithstanding—*dis*trust.

Thinking back to cases where B offers a narrative contribution about some aspect of A’s autobiography which, in some respects they are epistemically well-equipped to do so relative to A. Perhaps B remembers something that happened to A which B interprets as significant, which A does not remember. That is not sufficient for narrative deference, *even if* B is trusted over many other domains. The difference-maker at play here is how much A trusts their own narrative contribution about *this* event and *relative to* B’s. A may trust B in various capacities, but trust their own narrative interpretation of some feature of their autobiographical self-narrative more than B’s.

Matters of (ii) are fluid, and often negotiated and renegotiated through the course of our interactions with others. Being open to narratively deferring to another need not even necessarily presuppose *harmony* between narrators; it only requires an openness to the possibility that their interpretation may be more fitting than my own. This seems to track typical characteristics of therapeutic relationships, close friendships and other close relationships: we might disagree, we might battle over interpretations, we might try to convince the other or resist being convinced of some interpretation. Here, trust in oneself is constantly under review in light of the others’ contribution.

## Narrative Deference and Harm

While self-trust can be undermined already by mnemonic impairment, self-trust can be further eroded by precisely the people we narratively defer to in order to supplement that impairment. In this section, I explore some of the ways in which narrative deference can cause, maintain and exacerbate low self-trust in one’s memories and autobiographical self-narrative. This provides a good foundation for explaining why, even in relationships infected with suspicion, resentment and indignation, narrative deference can still occur.

My analysis of narrative deference so far has made clear the ways in which it is good for us: we generally seek to protect ourselves by exercising discernment in who we take to be epistemically credible i.e. worth deferring to. However, more careful consideration of the idea that low self-trust can dispose a person towards narrative deference, even if they are suspicious or distrustful of the person offering a narrative of some aspect of their autobiographical self-narrative, uncovers additional layers of complexity. In what follows, I intend to illustrate the messiness of the boundary between narrative deference as elective versus imposed, and as beneficial versus harmful.

We can begin by noting that not all close personal relationships are straightforwardly chosen: it is a fact, for instance, that we cannot chose our birth parents, who are often our primary caregivers. Some family dynamics may involve the imposition of certain narratives which can involve complex combinations of benefit and harm. While a person can be hermeneutically enabled by bringing certain experiences to narrative order with the help of another person, they can also be unjustly harmed if that narrative denies them certain resources or goods which it is in their best interests to have. Drawing on the notion of epistemic injustice—where a person is harmed in their capacity as a knower—we might consider such a person to be *hermeneutically marginalised* if they are victim to a dynamic whereby only the dominant interpretation of their experience is accepted as true (Fricker 2007). We can, I suggest, make sense of some of the harms of narrative deference in close relationships through this lens. Consider Case 1 discussed in Sect. 3, where a young male victim of abuse by his father later narratively defers to his mother about how the abuse has affected his character. His mother’s narrative interpretation is dominant, and it may help the boy in various ways to give narrative order to experiences which might otherwise evade it. Yet, its contents may simultaneously deprive the boy of useful hermeneutic resources. For instance, his mother may attribute features of her son’s behaviour or character to his father’s “bad influence”, an interpretation of hers which may be significantly influenced by her own trauma and autobiographical self-narrative (Schechter et al. 2008).^11^

We may also find ourselves pulled between a plurality of self-narratives depending on the interpersonal situation we find ourselves in. Consider children who are under divided custody of separated parents: they may be exposed to different autobiographical self-narratives which, in different contexts, are dominant. In many cases like these, a caregiver will be sincere and benevolent in their contribution to their child’s autobiographical self-narrative, despite the fact that adoption of that narrative may deny them certain hermeneutical resources with which they might better understand their own experience. While matters are complicated, dependence upon the sincere but impoverished narrative contributions of others can be, in principle, distinguished from dependence upon narrative contributions driven by manipulation. Consider the following quote from Whitney, in the context of her work on *affective* injustice:

On the interpersonal scale of relationships among individuals, sharing affective influence on each other is a necessary ingredient of intimacy: to be “close” to you, not in the spatial but the emotional sense, is to be inclined to give your emotions uptake, where the degree of closeness can be measured in the degree of unconditional uptake you enjoy from me. To be sure, affective influence can be abused—indeed, I would think taking advantage of such trust is constitutive of emotional varieties of abuse. (Whitney 2023a, p.1274)

Whitney connects affective injustice to a distinctively affective form of *gaslighting*, the phenomena whereby casting doubt on someone is the means through which they are made to doubt themselves; “in gaslighting, the way others respond to me begins to impair an aspect of my relationship to myself. In particular, the noncooperative response I receive from some second person(s) when I express my experiences to them begins to impair some aspect of my ability to make sense of my own experience to myself in the first person” (Whitney 2023b, pp.31–32). Understood this way, we can see how the affective influence others have on us can be used to undermine our self-trust over time such that our autobiographical self-narrative possibilities are restricted. While gaslighting is primarily discussed in the context of romantic relationships, one can also be a victim of this at the hands of family, friends, colleagues, healthcare professionals, religious figures and so on. It need not be the case, I suggest, that A only narratively defers if A soberly judges B’s interpretation about some autobiographical event of A’s to be more fitting than A’s. There are a range of more complex considerations at play which allow us to make sense of the different motivations involved in narratively deferring.

For instance, one can narratively defer to another because it presents itself as the best immediate option in order to sustain one’s autobiographical self-narrative. This might be so because it allows one to preserve a particular good, or realise certain positive, grounding, relieving or safety-preserving affective states. Here the niche construction framework is helpful in pointing towards different instrumental reasons for engaging in narrative deference with certain people, in certain environments. This is usefully cashed out by Coninx in her discussion of “inter-scale conflicts” whereby certain environmental conditions constructed by the organism appear adaptive on one spatio-temporal scale but maladaptive concerning another (Coninx 2023). Ontogenetic niche construction refers to an individual’s idiosyncratic engagement with their environment over time, which allows them to build stable patterns of engagement with their environment to support their well-being. On the other hand, microgenetic niche construction is concerned with obtaining physical, cognitive, or affective goods in the “here and now”. (pp.3009–3010). Coninx describes ontogenetic—microgenetic scale conflicts as follows:

Concerning this potential conflict, we might think of environmental modifications that prove beneficial for an organism’s general wellbeing but complicates or hinders their problem-solving in a particular context. In a nutshell, a person’s overall interests are not always compatible with their local interests. For example, actively constructed social relationships that provide a sustainable source of support might in concrete situations prevent an agent to use environmental structures most efficiently. Imagine an old couple relying on each other in regulating emotions or memorizing past events (Sutton, 2010). Overall, such relation is beneficial for both partners from an ontogenetic perspective while this might still lead to poorer performance in situations in which they have to face corresponding challenges in isolation. (Coninx 2023, p.3016)^12^

The desire to preserve intimacy with another person can be a motivational factor involved in narrative deference. In their work on interpersonal affective scaffolding, consider Colombetti & Krueger’s remark that “part of the reason we experience intimacy with family and friends is because we know what sort of affective feedback we can expect from them.” (Colombetti and Krueger 2015). Microgenetically, a sense of intimacy can be highly adaptive. Yet, the preservation of intimacy can come at some cost if it makes one lesser-equipped to pursue a more authentic autobiographical self-narrative.

For instance, A might lack the self-trust to reject features of their self-narrative proposed by B which ascribes false motivations to A (perhaps B proposes, wrongly, that A was sexually assaulted because A somehow invited it). Adopting such interpretations into one’s autobiographical self-narrative may not have the quality of “feeling right” or “making sense”, and they are almost certainly not ontogenetically adaptive. Yet, there may be (i) no felt prospect of a viable alternative on the horizon and (ii) a risk that rejecting the narrative will undermine the intimacy between A and B, leaving A socially isolated.

Recall that as well as by explicitly offering narrative contributions conversationally, interlocutors can influence the unfolding of a self-narrative by a range of other more subtle paralinguistic means (Fabry 2023b). Various micro-influences can function to restrict the felt salience of an unfolding autobiographical self-narrative over time, significantly influencing how it continues. This might involve, for instance, subtle mocking and undermining gestures like smirks, eye-rolls, eyebrow raises and other expressions of incredulousness. An unfolding narrative can even be influenced by the very absence of gestures: silence. If one fails to get feedback on a tentative self-narrative, trust in that narrative can be further eroded, further undermining the person’s sense of agency and self-trust required to author their own autobiographical self-narrative and feeding their felt need to rely on others to construct it.

## Conclusion

In this paper, I have presented the concept of narrative deference, roughly understood as the phenomenon whereby A is more dependent upon B for central aspects of A’s autobiographical self-narrative than on A. I have suggested some background structures that can dispose a person towards narratively deferring to another, namely mnemonic impairment about some autobiographically significant adverse life event(s). I then discussed whether and how trust is implicated in relationships where narrative deference is experienced as possible or even necessary, and argued that a thin notion of trust is necessary for narrative deference. I then discussed some of the ways in which the harms and benefits of narrative deference are complexly related.

Since this paper is an initial proposal of the concept, I have limited my focus in this paper to dyadic relations (such as between close friends, parents and children, patients and therapists, and romantic partners). However, future research would surely benefit from considering how narrative deference can be (i) shaped by structural factors and (ii) occur at a structural level. This may connect the concept of narrative deference up with wider literature on various forms of structural oppression, as well as testing the limits of the concept characterised as *deference* i.e. chosen. For example, it is plausible that various instances of narrative deference can be caused by forms of emotional imperialism: “Emotional imperialism involves a powerful group imposing aspects of its culture’s emotional norms and standards on another less powerful group whilst at the same time marking out the other culture’s emotional norms and standards as deviant and inferior.” (Archer and Matheson 2022).

Second, I suggest this that the notion of narrative deference might also usefully feature in causal explanations of psychopathology following the death or separation from a loved one. For instance, rates of Prolonged Grief Disorder are higher amongst those identified as having been highly dependent upon, or anxiously attached to, the person who has died (Bowlby 1980; Field and Sundin 2001; Johnson et al. 2007; Russ et al. 2022). It could be informative to explore how patterns of narrative deference might be aetiologically relevant here.

## Footnotes

1 In order to capture the affective and autobiographical features of evocative objects, Heersmink also takes less tangible ‘objects’ like olfactory objects, music, images and stories to be evocative objects.

2 Typically, the theoretical frameworks of niche construction, distributed cognition/affectivity and scaffolding have different foci and explanatory scope. The cognitive/affective scaffolding literature, while narrower in scope, presupposes understanding cognition and/or affectivity as distributed because extra-neural resources are required to realise certain cognitive and/or affective states. In this paper, I follow Heersmink (2020) in his understanding of the relationship between distributed cognition and niche construction at play, namely that our memory ecologies are distributed, and we can engage with our memory ecologies in order to construct and maintain various niches and enjoy practical and evolutionary benefits from doing so. Niche construction can be understood in subtly different ways at different spatio-temporal scales (see helpful context in Fabry 2021 and Coninx 2023).

3 We can remember our personal past at different levels of abstraction, from periods of our lives perhaps spanning over a few years, to specific moments. We can remember facts and propositions, as well as phenomenologically-rich, affect-laden experiences. Moreover, the distinction between the two (sometimes understood as the distinction between episodic and semantic memory) can shift over time (Conway and Pleydell-Pearce 2000; Fabry 2023b).

4 It is worth noting that there are many significantly different views about the extent to which narrative is constitutive of, and involved in shaping, ‘self’. See Zahavi (2018) and Strawson (2004) for very influential yet entirely non-narrative understandings of the self, Gallagher (2013) for a pluralistic approach, and Hutto (2016) for a useful overview of the conceptual space at play here.

5 As far as I can tell, even though Heersmink adopts the self-constitution view, he does not commit himself to the view that the narrative self is the most fundamental aspect of self or indeed sufficient for the self to be realised; rather, he sees the postulation of narrative self as consistent with the minimal self as postulated by Zahavi (Heersmink 2020; Zahavi 2007). Perhaps Heersmink would like to ultimately subscribe to a more ‘pluralistic’ view of self as suggested by Gallagher’s pattern theory of self (2013). However, it is not clear how the minimal self, in the way that Zahavi intends it to be considered, is compatible with Gallagher’s pattern theory.

6 My account, therefore, is concerned with a very specific type of narrative i.e. self-narrative specifically defined. There are countless other types of narrative. How one should understand ‘narrative’ simpliciter is an open question. Lamarque (2004), and Ratcliffe and Byrne (2022) following him, take the position that the notion of narrative needs further qualification in order for it to be informative. Lamarque (2004) adopts a minimal notion of narrative, requiring only that they are told rather than discovered, are made up of at least two non-logically related events, and have a temporal structure.

7 Note that in narrative theory, it is typical to distinguish between the author and the narrator (e.g. see Lamarque 2014, p.54). Similarly, narrative can involve the interplay of many perspectives, internal and external, as discussed by Goldie in his work on free indirect style (2012 p.65). As one reviewer helpfully points out, one might wish to make a similar distinction here between the author of one’s self-narrative and the narrator of it. I suggest that the authoring role might be just as complicated as the narrating role, such that authorship and narration can be very strongly distributed between people.

8 It has been suggested that how adults who experienced trauma as children encode subsequent stressful experiences is affected by their earlier trauma since it reactivates memories of earlier experiences which were processed in a data-driven way (Ehlers and Clark 2000).

9 I limit my focus to the study of dyadic relationships here, but I am sure that there is scope to explore these dynamics on a variety of group levels from triads (such as parent unit-child relationships) to workforces or perhaps governments.

10 Køster takes this position to be broadly compatible with Meyers’ view that a narrative of “psychocorporeal meaning” will inevitably fall short (2014) and Porter Abbott’s contention that so-called “emergent processes” are unnarratable (2008).

11 Arguably, one of the central values of psychotherapy and counselling is in being professionally supported in “unpicking” such harms and opening up narrative possibilities. However, clients are also vulnerable to harmful effects of narrative deference at the hands of therapists and other healthcare practitioners (for instance, see Bacon 2024).

12 The conflict can also function the other way around, where ways of engaging with one’s environment can seem adaptive microgenetically despite being maladaptive ontogenetically e.g. conflict avoidance (2023).
